# Effects of moringa polysaccharides on growth performance, immune function, rumen morphology, and microbial community structure in early-weaned goat kids

**DOI:** 10.3389/fvets.2024.1461391

**Published:** 2024-11-06

**Authors:** Jinyang Liu, Jinyu Chen, Sicheng Fang, Baoli Sun, Yaokun Li, Yongqing Guo, Ming Deng, Duoen Zhou, Dewu Liu, Guangbin Liu

**Affiliations:** ^1^College of Animal Science, South China Agricultural University, Guangzhou, China; ^2^Guangdong Leader Intelligent Agriculture Co., LTD, Qingyuan, China

**Keywords:** moringa polysaccharide, goat kid, immune function, rumen morphology, rumen microbiota

## Abstract

The aim of this research was to investigate the effects of adding moringa polysaccharides (MOP) on the growth performance, immune function, rumen tissue morphology, and rumen microbial community in early-weaned goat kids. Twenty-one 7-day-old Leizhou male goat kids weighing (3.05 ± 0.63) kg, were randomly divided into a control group (CON group), a low-dose group (LOW group), and a high-dose group (HIG group). MOP was added to the goat kids’ milk replacer (MR) at 0, 0.15, and 0.3% (on dry matter basis),fed until 60 days of age, and four goat kids in each group with body weights close to the mean of each group were selected for slaughter. The results showed that, compared to the CON group, the MOP groups significantly improved final body weight, body measurements, daily weight gain, and feed intake of the early weaned goat kids; significantly reduced the content of propionic acid, butyric acid, valeric acid, and ammoniacal nitrogen; and in addition, the addition of MOP could significantly increase the height of rumen nipple, the content of immunoglobulin G (IgG) in the serum. The HIG group significantly increased rumen pH, rumen muscularis layer thickness, rumen wall thickness, and serum immunoglobulin A (IgA), and immunoglobulin M (IgM). In conclusion, the addition of MOP positively impacted the growth performance, serum immune function, and rumen tissue morphology in early-weaned goat kids.

## 1 Introduction

As a unique digestive organ of ruminants, the development of rumen plays an important role in the health and digestive function of young ruminants. Early weaning of lambs can shorten the reproductive cycle of ewes and increase lambing frequency (1), and at the same time promote the development of rumen structure and function (2, 3). However, mother lamb separation and changes in feed type can easily cause stress in early weaned lambs (4), which not only reduces the immunity and growth rate of lambs, but also results in the underutilization of nutrients and blockage of the immune system during the weaning period, which can easily lead to diseases such as intestinal pathogenic bacterial infections, intestinal homoeopathy imbalance and diarrhea, and even cause death (5–7). Antibiotics have been widely used to treat and prevent diarrhea in lambs (8), but the frequent use of antibiotics not only causes great damage to the balance of intestinal flora and immune system in lambs, but also the antibiotic residues in the intestinal tract and feces of the animals lead to the increased risk of bacterial microorganisms’ resistance, and these antibiotic residues of fecal matter and animal products can cause great negative impacts on human beings and the ecological environment (9). Under the Chinese government’s antibiotic ban, finding natural active substitutes for antibiotics to achieve healthy lamb farming is of great importance for the high-quality development of the goat industry.

Moringa is a perennial deciduous tree of the Moringaceae family, native to India, and nowadays it has been widely cultivated as a multipurpose plant in different countries and regions (10, 11). All parts of Moringa including leaves, rhizomes and seeds are rich in functional polysaccharides, which are not only of high nutritional value but also of medicinal value (10). The presence of ascorbic acid, carotenoids and flavonoids, phenols and other types of antioxidant compounds in the leaves of Moringa is a good source of natural antioxidants (12). Polysaccharides extracted from Moringa are highly bioactive and have anti-inflammatory, antioxidant, antimicrobial, immunomodulatory and gastrointestinal protective properties (13). Natural plant polysaccharides have strong immunomodulatory functions and are considered as ideal drugs to improve immunity and antitumor (14–16). The experimental results of Tian et al. (17) have showed that MOP enhanced immunity, improved intestinal flora and morphology, as well as modulated the metabolism in mice. Zhao et al. (18) demonstrated that MOP could improve calf diarrhea, improve immunity, improve intestinal flora as well as promote growth.

Therefore, we added different concentrations of MOP to explore its effects on growth performance, serum antioxidant capacity, immune capacity, gastrointestinal morphology and microbial composition of 7-day-old weaned goat kids. In order to evaluate the feeding value of MOP in early-weaned goat kids and determine the appropriate addition amount of MOP in the actual production of goat kids, it provides a theoretical basis for the more scientific and rational application of MOP in goat industry.

## 2 Materials and methods

### 2.1 Ethics statement

All experimental procedures in this study were approved by the Committee of Animal Experiments of South China Agricultural University (No. 2023g032).

### 2.2 Experimental design and treatments

The feeding experiment was conducted from November 2023 to January 2024 at a black goat farm in Qingyuan, Guangdong Province. During the trial period, the average temperature was 16.4°C (ranging from 5 to 28.5°C) and the average humidity was 67.1% (ranging from 22 to 93%).

MOP was purchased from Xi’an Clover Biotech Co., Ltd. (Xi’an, China), brown powder with 70% purity. Milk replacer (MR) was purchased from Beijing Precision Animal Nutrition Research Center (Beijing, China). Nutritional levels (dry matter basis): crude protein 27%, crude fat 18%, crude ash 10%, calcium 1.5%, phosphorus 1.2%. The starter diet was a granular compound feed formulated with reference to the nutritional requirements of NRC (1994), and the composition and nutritional levels of the basal ration are shown in Table 1.

**Table 1:** Composition and nutritional levels of the starter feed (air-dry basis).

Ingredients	Composition (%)
Corn	46.40
Soybean meal	15.60
DDGS	10.35
Dried whey	20.25
CaHPO_4_	1.00
Limestone	0.81
NaCl	4.09
Choline chloride	0.50
Premix	1.00
Total	100.00

Twenty-one 7-day-old Leizhou goat male goat kids of close weight and good health were selected with an average initial weight of (3.05 ± 0.63) kg. The goat kids were randomly divided into three groups of seven replicates of one goat kid.

The amount of MOP added was referred to the experimental results of Guo et al. (5). The control group (CON group) was fed milk replacer. The low dose group (LOW group) was fed milk replacer supplemented with 0.15% of their daily dry matter intake (DMI) of MOP. The high dose group (HIG group) was fed milk replacer supplemented with 0.3% of their daily dry matter intake (DMI) of MOP. The goat kids were fed from 7 days of age to 60 days of age, with a total experimental period of 54 days.

### 2.3 Feeding management

The test goat kids were suckled with their mothers until 7 days of age, and were forced to wean at 7 days of age. The goat kids were fed in separate pens (3.5 m long, 2.5 m wide and 1.5 m high), with partitions between the pens to prevent the groups from coming into contact with each other. Each pen is equipped with a drinking trough and trough for goat kids to freely eat open food, hay and sufficient water. The troughs were cleaned and replaced every morning. Straw is used as bedding for goat kids to lie on and is replaced every 2 days. All goat kids were provided with free access to starter feed and received an equal amount of milk replacer powder (2% of body weight) daily at 08:00, 12:00, and 16:00. The milk replacer powder was reconstituted with warm water at a 1:7 (weight/volume) ratio, thoroughly mixed until fully dissolved, and then dried at room temperature to 40°C. The reconstituted milk replacer was administered via a 250 mL bottle.

The sanitation of the pens was carried out according to the management measures of the farm, and was cleaned and disinfected regularly. The goat pens were cleaned and disinfected regularly according to the farm management measures, and the goat were immunized according to the normal immunization procedures.

### 2.4 Experimental methods and parameter measurements

#### 2.4.1 Feed intake measurement

Feed samples, including hay, milk replacer, and starter, were collected weekly during the trial and mixed at the end of the trial. The feed intake of each group of goat kids was recorded daily. The collected feed samples were oven dried at 65°C to constant weight, pulverized and then determined for dry matter, crude ash, crude protein, crude fat, acid detergent fiber, neutral detergent fiber, calcium and phosphorus with reference to the assay method of Horwitz et al. (19).

#### 2.4.2 Growth performance measurement

Goat kid fasting weight and body measurements were determined at the beginning of the trial (i.e., when goat kids were 7 days old), and at the end of the trial (i.e., when goat kids were 60 days old) prior to morning feeding, and the average daily weight gain of the test goat kids was calculated.

#### 2.4.3 Blood sample collection and analysis

Blood was collected from the jugular vein of the goat kids before morning feeding on the 60th day of the experiment using a procoagulant blood collection tube, centrifuging at 3,000*g* at 4°C for 15 min, and the serum was centrifuged and collected for the determination of biochemical, antioxidant, and immunological indices. Total protein (TP), albumin (ALB), globulin (GLB), urea nitrogen (BUN), glucose (GLU), triglyceride (TG), total cholesterol (TC), high-density lipoprotein (HDL) cholesterol, low-density lipoprotein (LDL) cholesterol, alanine aminotransferase (ALT), alanine oxalate aminotransferase (AST), and creatinine (CRE) were measured by using an automated biochemistry instrument (Zecheng CLS880); total antioxidant capacity (T-AOC) in serum was determined by 2,2′-azino-bis(3-ethylbenzothiazoline-6-sulfonic acid) (ABTS) method (Item No.: A015-2-1); catalase (CAT) activity was determined by ammonium molybdate method (Item No.: A007-1-1); and superoxide dismutase (SOD) activity was determined by Water-Soluble Tetrazolium Salt-1(WST-1)method (Item No.: A001-3-2); Glutathione peroxidase (GSH-Px) activity (Cat. No.: A005-1-2); Malondialdehyde (MDA) content was determined by Thiobarbituric Acid (TBA) method (Cat. No.: A003-1-2); Serum immunoglobulin A (IgA), immunoglobulin G (IgG), immunoglobulin M (IgM) were determined by enzyme-linked immunosorbent assay (ELISA) kit; Serum The levels of pro-inflammatory factor interleukin-2 (IL-2), anti-inflammatory factor interleukin-6 (IL-6), tumor necrosis factor (TNF-*α*) and *γ*-interferon (IFN-γ) in serum were determined by using the kit of Nanjing Jianjian Institute of Biological Engineering, and the absorbance was measured by using an enzyme labeling instrument (RaytoRT-6100), and the specific procedures were carried out according to the instructions of the kit.

#### 2.4.4 Collection and analysis of rumen tissue and rumen contents

Goat kids were euthanized and dissected for sampling within 15 min. Post-dissection rumen pH was determined using a pH meter (FE28, METTLER TOLEDO INSTRUMENTS CO., LTD., Shanghai, China). Goat kid rumen content samples were stored in liquid nitrogen for subsequent DNA extraction. Rumen fluid samples obtained by filtration using four layers of gauze were stored at −20°C for subsequent determination of fermentation parameters.

The rumen ventral sac tissue (1.5 × 1.5 cm^2^) of goat kids was collected after autopsy with reference to the method of Li et al. (20), and after rinsing with phosphate buffer solution (PBS), the rumen tissue was fixed for 24 h in 4% paraformaldehyde solution, dehydrated, transparent, and wax impregnated to obtain paraffin-embedded wax blocks of rumen tissue.

### 2.5 Morphology and measurement of rumen tissue

The embedded wax block was cut into 4 μm thick slices with a paraffin slicer (RM2016), spread horizontally under 40°C warm water in a spreader, and the slide fished the tissue and baked the slices in a 60°C oven. After the water baking dry wax baked and removed, the slices were sequentially put into environmentally friendly dewaxing solution I 20 min—environmentally friendly dewaxing solution II 20 min—anhydrous ethanol I 5 min—anhydrous ethanol II 5 min—75% alcohol for 5 min, and washed with tap water. The slices were put into HD constant dye pretreatment solution for 1 min, hematoxylin dyeing solution for 3-5 min, washed with tap water, differentiation solution for differentiation, washed with tap water, return blue solution for return blue, rinsed with running water; dehydrated with 95% alcohol for 1 min, and stained into eosin dyeing solution for 15 s; anhydrous ethanol I2min—anhydrous ethanol II2min—anhydrous ethanol III2min—n-butanol I2min—n-butanol II2min-dimethyl I2min-xylene II2min transparent, neutral gum sealing, microscopic observation of histological morphology and scanning and photographing using panoramic scanner scanning software. The rumen papillae, papillae width, muscular layer thickness, rumen wall thickness, cuticle thickness and calculated papillae density, rumen papillae specific surface area using (Image-ProPlus6.0, United States) analysis software, one field of view was selected for each section, and histomorphology was counted in each field of view.

### 2.6 Measurement of rumen fermentation parameters

The concentrations of acetic acid (AA), propionic acid (PA), butyric acid (BA), valeric acid (VA), isobutyric acid (IBA), and lactic acid (LA) were detected by gas chromatography (Agilent 7890B, NYSE: A, Palo Alto, CL, United States) with reference to the method of Wang et al. (21). A total of 0.2 mL of 25% metaphosphoric acid was added to 1 mL of rumen fluid sample and mixed properly. Subsequently, the mixed samples were stored at −20°C for more than 24 h. The samples were then centrifuged at 10,000 rpm for 10 min at 4°C, and 1.0 mL of the supernatant was filtered through a 0.45 μm membrane. The filtrate was then injected into special gas phase vials where 0.4 μL of the sample was automatically injected into the HP-INNOWax gas phase capillary column. The injector and detector temperatures were set to 250°C and 280°C, respectively, and the split ratio was set to 40:1. The column was heated from 120°C to 250°C at 10°C/min. Ammonia-nitrogen concentration (NH_3_-N in rumen fluid) was determined by a colorimetric method as described by Ma et al. (22).

### 2.7 Determination of rumen microbial flora in rumen contents

Total genomic DNA samples were extracted using the OMEGA Soil DNA Kit (M5635-02) (Omega Bio-Tek, Norcross, GA, United States), following the manufacturer’s instructions, and stored at −20°C prior to further analysis. The quantity and quality of extracted DNAs were measured using a NanoDrop NC2000 spectrophotometer (Thermo Fisher Scientific, Waltham, MA, USA) and agarose gel electrophoresis, respectively. PCR amplification of the bacterial 16S rRNA genes V3–V4 region was performed using the forward primer 338F (5’-ACTCCTACGGGAGGCAGCA-3′) and the reverse primer 806R (5’-GGACTACHVGGGTWTCTAAT-3′). Sample-specific 7-bp barcodes were incorporated into the primers for multiplex sequencing. The PCR components contained 5 μL of buffer (5×), 0.25 μL of Fast pfu DNA Polymerase (5 U/μL), 2 μL (2.5 mM) of dNTPs, 1 μL (10 uM) of each Forward and Reverse primer, 1 μL of DNA Template, and 14.75 μL of ddH2O. Thermal cycling consisted of initial denaturation at 98°C for 5 min, followed by 25 cycles consisting of denaturation at 98°C for 30 s, annealing at 53°C for 30 s, and extension at 72°C for 45 s, with a final extension of 5 min at 72°C. PCR amplicons were purified with Vazyme VAHTSTM DNA Clean Beads (Vazyme, Nanjing, China) and quantified using the Quant-iT PicoGreen dsDNA Assay Kit (Invitrogen, Carlsbad, CA, United States). After the individual quantification step, amplicons were pooled in equal amounts, and pair-end 2 × 250 bp sequencing was performed using the Illlumina NovaSeq platform with NovaSeq 6,000 SP Reagent Kit (500 cycles) at Shanghai Personal Biotechnology Co., Ltd. (Shanghai, China). The original 16S rRNA sequence data has been submitted to NCBI with the accession number PRJNA1159108.

### 2.8 Data processing and statistical analysis

Experimental data were organized and calculated using Excel 2019. Statistical analysis of the data was performed using SPSS 23.0 with one-way analysis of variance (ANOVA), and the results were expressed as means and standard error of the mean (SEM). Duncan’s method of multiple comparisons was used for the test of variance, and *p* < 0.05 was used to indicate significant differences.

## 3 Results

### 3.1 Impact of moringa polysaccharides on the growth performance of goat kids

As shown in Table 2, initial body weight and milk replacer intake did not differ significantly among groups (*p* > 0.05); however, final body weight, average daily gain, openings, and green hay intake were significantly higher in the LOW and HIG groups compared to the CON group (*p* < 0.05).

**Table 2:** Effects of moringa oligosaccharides on the growth performance of early weaned goat kids.

Items	CON	LOW	HIG	SEM	*p*-Value
Initial BW (kg)	3.01	3.06	3.09	0.14	0.976
Final BW (kg)	5.40^b^	6.95^a^	7.36^a^	0.30	0.009
ADG (g/d)	44.96^b^	73.45^a^	80.54^a^	4.92	0.020
Milk replacer intake (g/d)	490.95	495.96	483.06	14.82	0.938
Starter intake (g/d)	284.11^b^	519.24^a^	582.39^a^	49.11	0.032
Green hay intake (g/d)	90.79^b^	165.29^a^	166.32^a^	10.68	0.003

CON, the milk replacer (n = 7); LOW, supplemented with 0.15% MOP in the milk replacer (n = 7); HIG, supplemented with 0.3% MOP in the milk replacer (n = 7); BW, body weight; ADG, average daily gain; $\bar{x}$ represents mean; SEM standard error of the mean. Same lowercase letters within the same row indicate no significant difference (*p* > 0.05), while different lowercase letters indicate a significant difference (*p* < 0.05). The same applies to the following tables.

### 3.2 Effect of moringa polysaccharides on serum biochemical parameters of goat kids

As shown in Table 3, the addition of MOP had no significant effect on GLU, TP, ALB, BUN, TG, TC, HDL, LDL, ALT, AST and CRE contents of goat kids (*p* > 0.05), in which the TG content showed a decreasing trend with the increase of MOP additions (*p* = 0.088); GLB content of the HIG group was significantly lower than that of the CON group (*p* < 0.05), the difference in GLB content between the LOW group and the CON and HIG groups was not significant (*p* > 0.05); the A/C ratio of the CON group was significantly lower than that of the LOW and HIG groups (*p* < 0.05).

**Table 3:** Effect of moringa polysaccharides on serum biochemical parameters of goat kids.

Items	CON	LOW	HIG	SEM	*p*-Value
GLU (mmol/L)	4.11	5.34	4.78	0.28	0.199
TP (g/L)	51.11	47.72	47.16	0.88	0.131
GLB (g/L)	32.81^a^	29.87^ab^	26.74^b^	1.05	0.028
ALB (g/L)	19.06	20.26	20.42	0.49	0.522
A/G	0.52^b^	0.71^a^	0.76^a^	0.04	0.021
BUN (mmol/L)	7.31	7.88	6.25	0.34	0.125
TG (mmol/L)	0.37	0.13	0.17	0.05	0.088
TC (g/L)	1.37	1.74	1.26	0.11	0.175
HDL (mmol/L)	0.97	0.97	1.01	0.04	0.930
LDL (mmol/L)	1.17	1.41	1.10	0.07	0.180
ALT (U/L)	27.83	28.19	31.41	0.78	0.105
AST (U/L)	106.54	90.40	92.87	3.61	0.137
CRE (μmol/l)	13.41	6.20	7.24	1.68	0.166

CON, the milk replacer (*n* = 3); LOW, supplemented with 0.15% MOP in the milk replacer (n = 3); HIG, supplemented with 0.3% MOP in the milk replacer (*n* = 3).

### 3.3 The effect of moringa polysaccharides on serum antioxidant indices in goat kids

As shown in Table 4, the addition of MOP had no significant effect on T-AOC and CAT contents of goat kids (*p* > 0.05); the GSH-Px content of the LOW group was significantly lower than that of the CON group (*p* < 0.05), and the difference was not significant compared with that of the HIG group (*p* > 0.05); and the contents of SOD and MDA in the LOW group and the HIG group were significantly lower than that of the CON group (*p* < 0.05).

**Table 4:** Effect of moringa polysaccharides on serum antioxidant indices in goat kids.

Items	CON	LOW	HIG	SEM	*p*-Value
T-AOC (U/L)	0.75	0.74	0.74	0.01	0.930
CAT (U/mL)	0.28	0.18	0.21	0.02	0.146
GSH-Px (mol/L)	105.67^a^	79.40^b^	97.91^ab^	4.76	0.036
SOD (U/mL)	195.34^a^	174.69^b^	172.11^b^	3.81	<0.001
MDA (nmol/mL)	2.55^a^	1.98^b^	2.02^b^	0.12	0.049

CON, the milk replacer (*n* = 3); LOW, supplemented with 0.15% MOP in the milk replacer (*n* = 3); HIG, supplemented with 0.3% MOP in the milk replacer (*n* = 3).

### 3.4 Effect of moringa polysaccharides on the content of serum immunoglobulins and cytokines in goat kids

As shown in Table 5, the effects of adding MOP on IFN-*γ*, IL-2 and IL-6 contents of goat kids were not significant among the groups (*p* > 0.05); the IgA and IgM contents of the HIG group were significantly higher than those of the CON group (*p* < 0.05), and the IgA and IgM contents of the LOW group were not significantly different from those of the CON and HIG groups (*p* > 0.05); the IgG and TNF-*α* content was significantly higher than that of CON group (*p* < 0.05), but the difference between the two groups was not significant (*p* > 0.05).

**Table 5:** Effect of moringa polysaccharides on the content of serum immunoglobulins and cytokines in goat kids.

Items	CON	LOW	HIG	SEM	*p*-Value
IgA (μg/mL)	34.38^b^	39.87^ab^	44.70^a^	1.70	0.013
IgG (μg/mL)	1568.44^b^	1932.73^a^	1987.27^a^	76.07	0.016
IgM (μg/mL)	17.61^b^	19.96^ab^	22.27^a^	0.84	0.046
IFN-γ (pg/mL)	42.85	40.70	41.21	1.02	0.728
IL-2 (pg/mL)	110.59	113.28	120.67	6.61	0.852
IL-6 (pg/mL)	249.33	240.37	244.36	5.20	0.825
TNF-α (pg/mL)	549.02^b^	972.87^a^	865.95^a^	67.58	0.001

CON, the milk replacer (*n* = 3); LOW, supplemented with 0.15% MOP in the milk replacer (*n* = 3); HIG, supplemented with 0.3% MOP in the milk replacer (*n* = 3).

### 3.5 The effect of moringa polysaccharides on rumen fermentation parameters in goat kids

Rumen fermentation parameters are summarized in Table 6. There were no significant differences among the Total VFA, AA, and IBA groups (*p* > 0.05). PA, BA, VA, and NH3-N in the CON group were significantly higher than those in the LOW and HIG groups (*p* < 0.05). The AA:PA ratio in the HIG group was significantly higher than in the LOW and CON groups (*p* < 0.05). The pH value of the HIG group was significantly higher than that of the CON group (*p* < 0.05), but there was no significant difference between the HIG and LOW groups (*p* > 0.05).

**Table 6:** Effect of moringa polysaccharides on rumen fermentation parameters in goat kids.

Items	CON	LOW	HIG	SEM	*p*-Value
pH	5.54^b^	5.83^ab^	6.01^a^	0.10	0.020
NH_3_-N (G/L)	214.69^a^	70.02^b^	75.02^b^	25.67	0.003
Total VFA (mmol/L)	327.13	148.74	204.09	41.32	0.206
Acetic acid (mmol/L)	96.12	61.64	139.92	27.94	0.564
Propionic acid (mmol/L)	169.87^a^	65.01^b^	33.55^b^	20.00	0.001
Acetate: Propionic	0.53^b^	1.36^b^	4.89^a^	0.68	0.004
Butyric acid (mmol/L)	38.13^a^	12.96^b^	19.94^b^	4.68	0.059
Valeric acid (mmol/L)	15.22^a^	5.50^b^	3.77^b^	1.98	0.019
Isobutyric acid (mmol/L)	7.79	3.63	6.90	0.85	0.103

CON, the milk replacer (*n* = 4); LOW, supplemented with 0.15% MOP in the milk replacer (*n* = 4); HIG, supplemented with 0.3% MOP in the milk replacer (*n* = 4).

### 3.6 Effect of moringa polysaccharides on the morphology of goat kid rumen tissue

**Figure 1:**
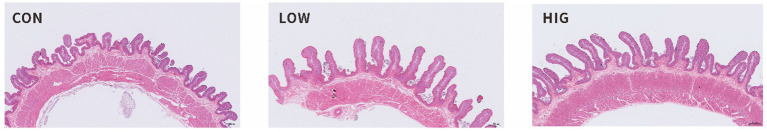
The histological sections of the rumen tissue in goat kids.

As shown in Figure 1, the CON group displayed short, thick, irregular, and untidy rumen papillae. In contrast, the rumen papillae in the LOW and HIG groups were elongated, neat, and relatively regular. The effect of moringa polysaccharides on the morphology of lamb rumen tissue is shown in Table 7. The differences among groups in papilla width, cuticle thickness and specific surface area of rumen papillae were not significant (*p* > 0.05); papilla height in the HIG and LOW groups was significantly higher than that in the CON group (*p* < 0.05); the thickness of the muscularis propria and the thickness of the rumen wall in the HIG group was significantly higher than that in the LOW and CON groups (*p* < 0.05); and the density of papillae in the CON group was significantly higher than that in the LOW and HIG groups (*p* < 0.05).

**Table 7:** Effect of moringa polysaccharides on the morphology of goat kid rumen tissue.

Items	CON	LOW	HIG	SEM	*p*-Value
Rumen papillae height (mm)	0.91^b^	1.39^a^	1.46^a^	0.05	0.000
Rumen papillae width (mm)	0.49	0.48	0.48	0.01	0.819
Rumen muscular layer thickness (mm)	1.05^b^	0.97^b^	1.38^a^	0.06	0.004
Rumen wall thickness (mm)	2.42^b^	2.70^b^	3.14^a^	0.09	0.003
Stratum corneum thickness (mm)	0.0248	0.0237	0.0281	0.00	0.285
Rumen papillae density (items/mm^2^)	2.12^a^	1.53^b^	1.48^b^	0.09	0.004
Rumen papillae surface area ratio	2.14	2.02	2.04	0.08	0.825

CON, the milk replacer (*n* = 4); LOW, supplemented with 0.15% MOP in the milk replacer (*n* = 4); HIG, supplemented with 0.3% MOP in the milk replacer (*n* = 4).

### 3.7 Effect of moringa polysaccharides on the microbial flora of goat kids

**Figure 2:**
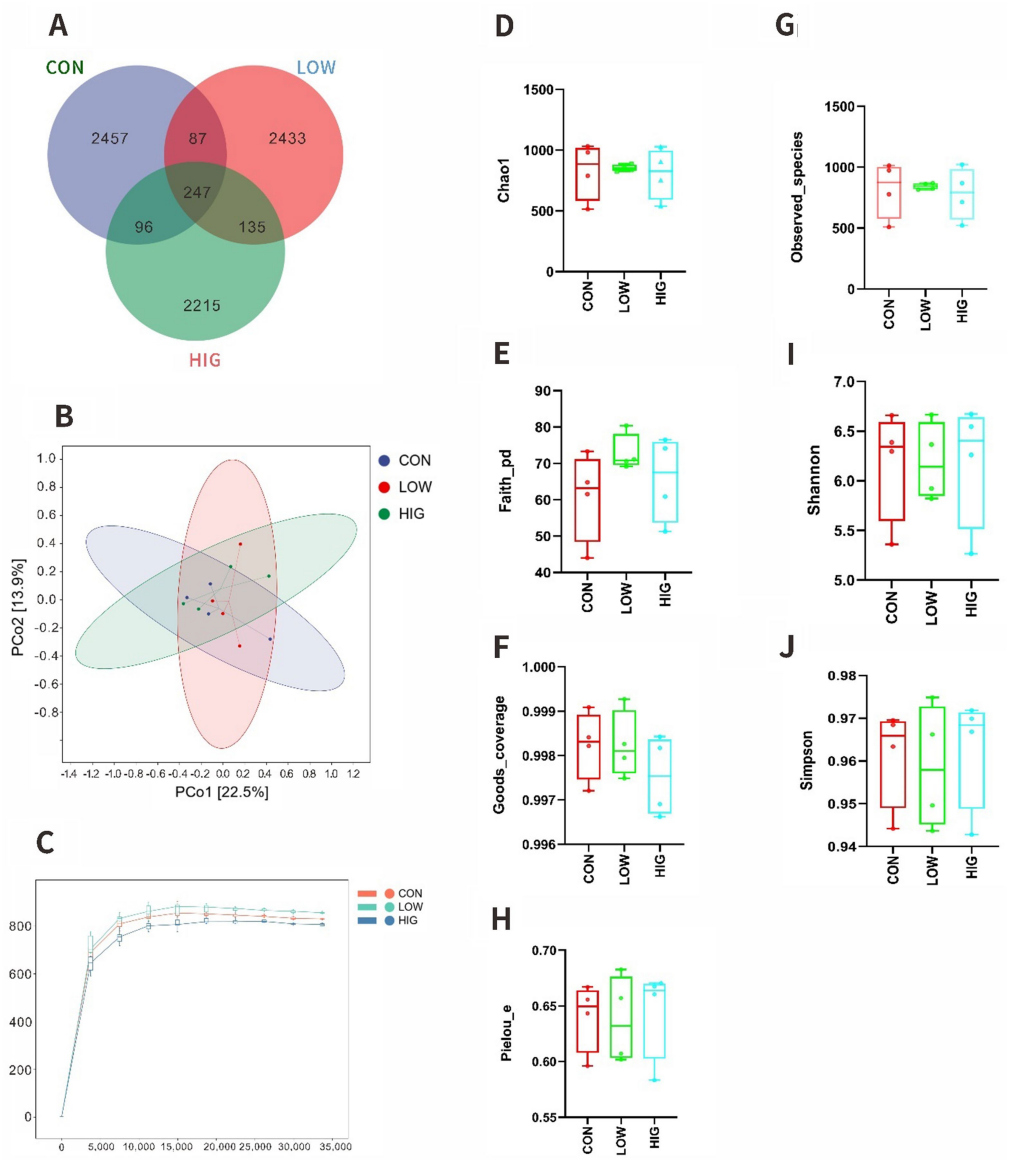
Effects of MOP on the alpha and beta diversity of the rumen microbiota in goat kids. Analysis of the alpha and beta diversity via **(A)** Venn figure of OTUs **(B)** Species observation curve in each group. **(C)** PCoA of weighted UniFrac distances, respectively **(D)** Chao1 index, **(E)** Faith_pd index, **(F)** Goods_coverage index, **(G)** Observed_species index, **(H)** Pielou_e index, and **(I)** Shannon index, **(J)** Simpson index; CON, the milk replacer (*n* = 4); LOW, supplemented with 0.15% MOP in the milk replacer (*n* = 4); HIG, supplemented with 0.3% MOP in the milk replacer (*n* = 4).

In this experiment, 12 samples of rumen contents from goat kids were sequenced and analyzed using IlluminaNovaseqMiseq platform, and a total of 944,860 original sequences were obtained, and quality control was performed on the obtained sequences, and a total of 878,895 valid sequences were obtained after removing low-quality, short-length, and chimerism. The valid sequences were clustered into OTUs according to 100% similarity, and a total of 7,670 OTUs were obtained, of which 247 OTUs were common to the three treatments, and 2,457, 2,433 and 2,215 OTUs were specific to the CON, LOW and HIG groups, respectively (Figure 2A). The observed curves of the species of the 12 samples are shown below, and the Rank Abundance curves reflect the groups’ Differences in species uniformity and abundance, as shown in Figure 2B, each sample was similar in uniformity and abundance, and the above results indicate that the sequencing depth of this study is sufficient and reasonable, and the results are stable and reliable, and can be used for subsequent analysis.

### 3.8 Effect of moringa polysaccharides on the alpha diversity of rumen microbiota in goat kids

Alpha diversity indices are commonly used to assess the diversity and richness of microbial species, and the value of Goods coverage reflects the coverage of OTUs in the samples, and the larger the value is, the more sufficient the sequencing data are, in this experiment, the value of Goods coverage of all the samples was not less than 0.996, which indicated that the depth of this sequencing could basically cover all the species. The results of the indexes in this experiment are shown in Figures 2D–J, and the alpha diversity index did not produce significant differences among treatment groups (*p* > 0.05).

### 3.9 Effect of moringa polysaccharides on the beta diversity of rumen microbiota in goat kids

PCoA is an unconstrained data dimensionality reduction method that reflects the similarity between samples based on their distribution distances on the graph. The closer the samples are on the graph, the more similar they are. NMDS analysis focuses more on the ordinal relationships between values and reflects the differences between samples based on the distances between points. In this test the CON group was not clearly distinguished from the other groups and the three groups overlapped each other (Figure 2C).

PERMANOVA analysis was further used in this trial to explore the beta diversity of rumen flora. The results are shown in Table 8, and the differences between the groups were not significant (*p* > 0.05).

**Table 8:** PERMANOVA analysis results.

Items	PERMANOVA		
	F	P	q
CON vs LOW	1.165	0.190	0.467
CON vs HIG	1.128	0.311	0.467
LOW vs HIG	0.795	0.705	0.705

CON, the milk replacer (*n* = 4); LOW, supplemented with 0.15% MOP in the milk replacer (*n* = 4); HIG, supplemented with 0.3% MOP in the milk replacer (*n* = 4).

### 3.10 Effect of moringa polysaccharides on the composition of rumen microbiota in goat kids

**Figure 3:**
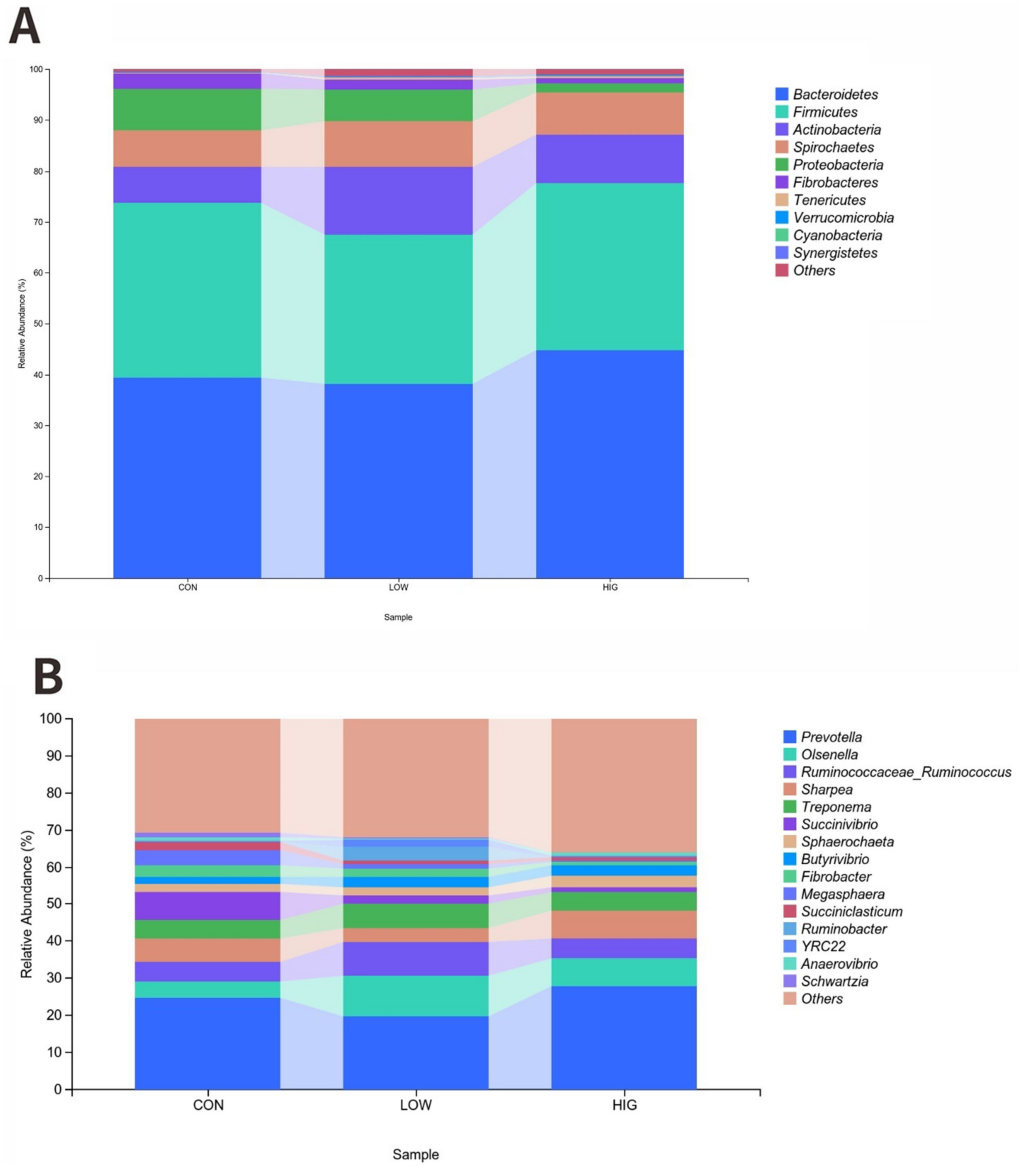
Effect of MOP on the composition of the rumen microbial community. **(A)** Phylum level **(B)** Genus level.

At the phylum level, Bacteroidetes and Fimicutes were the two main phyla, accounting for more than 67% of the rumen flora of goat kids (Table 9). The HIG group increased the relative abundance of Bacteroidetes by 5.4% compared to the CON group, but there was no significant difference at the phylum level of the phyla between the groups’ differences (*p* > 0.05) (Figure 3A).

**Table 9:** Effect of MOP on the relative abundance of rumen microbiota at the phylum level (%).

Items	CON	LOW	HIG	SEM	*p*-Value
Bacteroidetes	39.44	38.10	44.84	3.04	0.676
Firmicutes	34.28	29.45	32.69	1.93	0.627
Actinobacteria	7.04	13.24	9.56	1.74	0.375
Spirochaetes	7.24	9.04	8.36	0.80	0.695
Proteobacteria	8.17	6.11	1.75	1.78	0.356
Fibrobacteres	3.02	2.04	1.03	0.80	0.638
Tenericutes	0.16	0.40	0.34	0.11	0.697
Verrucomicrobia	0.06	0.14	0.28	0.09	0.645
Cyanobacteria	0.02	0.02	0.04	0.00	0.457
Synergistetes	0.02	0.03	0.02	0.00	0.344
Others	0.54	1.43	1.02	0.33	0.595

CON, the milk replacer (*n* = 4); LOW, supplemented with 0.15% MOP in the milk replacer (*n* = 4); HIG, supplemented with 0.3% MOP in the milk replacer (*n* = 4).

At the genus level, the top 15 abundant genera were *Prevotella, Olsenella, Ruminococcaceae_Ruminococcus, Sharpea, Treponema, Succinivibrio, Sphaerochaeta, Butyrivibrio, Fibrobacter, Megasphaera, Succiniclasticum, Ruminobacter, YRC22, Anaerovibrio,* and *Schwartzia*. The relative abundance results are shown in Table 10, the relative abundance of *Olsenella* genus. Increased in the high LOW group compared to the CON group, but there was no significant difference in the bacterial flora between the groups at the genus level (*p* > 0.05) (Figure 3B).

**Table 10:** Effect of MOP on the relative abundance of rumen microbiota at the genus level (%).

Items	CON	LOW	HIG	SEM	*p*-Value
*Prevotella*	24.88	19.71	27.82	1.91	0.228
*Olsenella*	4.09	11.05	7.58	1.44	0.140
*Ruminococcaceae_Ruminococcus*	5.39	9.06	5.24	1.20	0.372
*Sharpea*	6.39	3.73	7.71	1.52	0.600
*Treponema*	5.03	6.71	4.98	1.05	0.780
*Succinivibrio*	7.50	2.08	1.11	1.52	0.188
*Sphaerochaeta*	2.21	2.32	3.38	0.73	0.802
*Butyrivibrio*	1.93	2.70	2.60	0.67	0.900
*Fibrobacter*	3.02	2.04	1.03	0.80	0.638
*Megasphaera*	4.02	1.31	0.32	1.34	0.552
*Succiniclasticum*	2.18	1.13	0.99	0.28	0.159
*Ruminobacter*	0.05	3.61	0.00	1.20	0.408
*YRC22*	0.38	1.96	0.29	0.51	0.356
*Anaerovibrio*	0.91	0.32	0.74	0.15	0.303
*Schwartzia*	1.22	0.13	0.19	0.34	0.365
*Others*	30.81	32.14	36.01	2.41	0.701

CON, the milk replacer (*n* = 4); LOW, supplemented with 0.15% MOP in the milk replacer (*n* = 4); HIG, supplemented with 0.3% MOP in the milk replacer (*n* = 4).

### 3.11 Stomach microbiota LEfSe analysis

**Figure 4:**
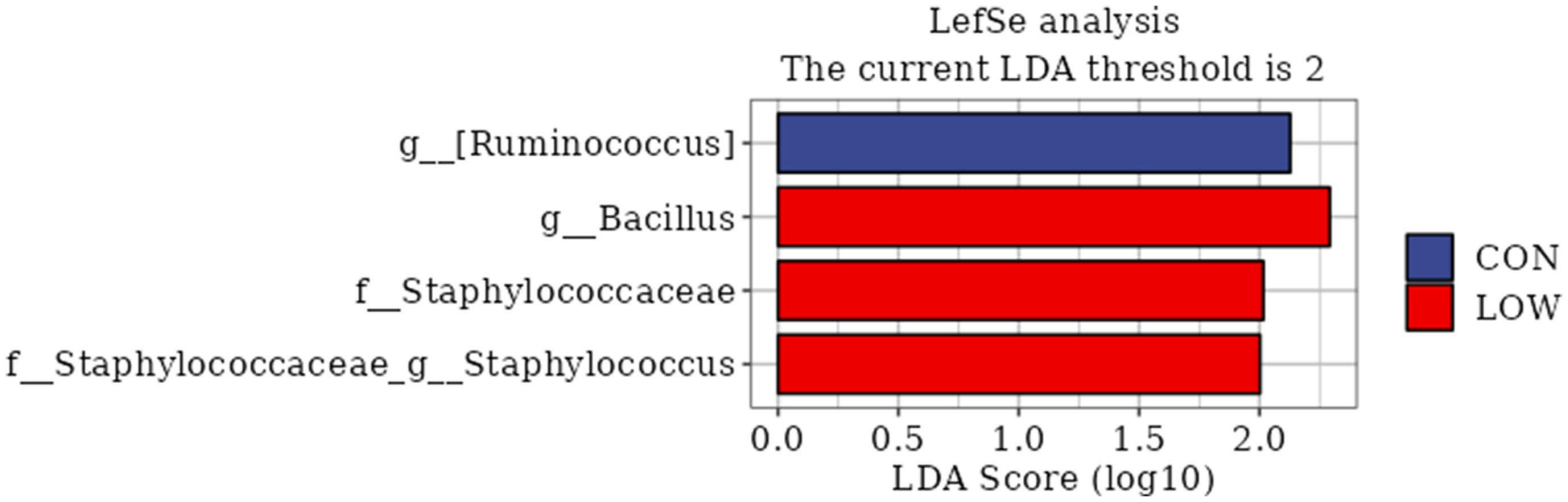
Bacterial taxa differences between control and LOW groups. Linear discriminant analysis (LDA) coupled with effect size (LEfSe) analysis was performed to identify the bacterial taxa differentially represented in control and LOW groups at different taxonomy levels. CON, the milk replacer (n = 4); LOW, supplemented with 0.15% MOP in the milk replacer (n = 4).

LEfSe analysis is a statistical method commonly used to analyze differences between groups, which not only enables comparisons between different subgroups, but also finds biomarkers that are statistically different. In this study, LDA > 2 was used as the threshold value, and four differential bacteria were screened, which were three families and one genus. Among them, the differential bacteria with large LDA values mainly include the *Ruminococcaceae* family, *Bacillus* genus, *Staphylococcus* genus, and *Staphylococcaceae* family (Figure 4).

## 4 Discussion

The rumen of ruminant animals plays a crucial role in nutrient digestion and absorption, immune response, and host metabolism (23). Ruminal development in ruminants at a young age is crucial and may have long-term effects on their health and later growth and development. According to Wang et al. (24) the addition of fermented wheat bran polysaccharides (FWBPs) to milk replacer increased daily weight gain and decreased feed to weight ratio in early weaned lambs. Chen et al. (25) added Chinese medicinal polysaccharides (CMPs) to increase the average daily weight gain of lambs and improved the lamb performance. Zhao et al. (18) added MOP to significantly increase the body weights of newborn calves. This is consistent with the significant increase in daily gain, body weight, and body size observed with MOP supplementation in this study. As the growth rate increases, nutritional demand also rises correspondingly. Additionally, in the MOP-added group, intake of green hay and other feeds increased significantly. This suggests that the improvement in growth performance is attributed to enhanced absorption and utilization of nutrients. Therefore, adding MOP can improve nutrient absorption and has a significant impact on promoting the growth and development of goat kids.

Changes in blood biochemical indicators can reflect alterations in the metabolic capacity of the organism. The serum A/G ratio and ALB content can reflect the protein synthesis of the organism, and the decrease of its ratio and content may be related to chronic inflammation caused by the accumulation of inflammatory cytokines (26, 27). The significant increase in the A/G ratio in this experiment indicated that the addition of MOP could reduce weaning stress in lambs and avoid stress-induced oxidative damage to proteins and lipids. Serum TG is a lipid metabolite (27). Addition of MOP in the present study reduced serum triglyceride levels in lambs. This is similar to the findings of Kwon et al. (28), which showed that plant polysaccharides such as MOP can affect lipid metabolism, leading to a decrease in serum TG content in goat kids. T-AOC, GSH-Px, SOD and other enzymes have important antioxidant functions, and T-AOC content can reflect the metabolic status of antioxidant free radicals, while MDA content can reflect the degree of oxidative stress, when the body undergoes oxidative stress, a large number of free radicals will be generated, and free radicals will react with lipids to produce MDA, which is the end product of lipid peroxidation, and the increase of MDA content represents the decrease of TG content. MDA is the end product of lipid peroxidation, and an increase in MDA content represents an increase in the level of oxidative stress in the body (29–31). In this study, there was no significant difference in the T-AOC content in the serum of goat kids among the groups, whereas the addition of MOP decreased the GSH-Px, SOD and MDA content in the serum of goat kids, which may be attributed to the binding of polysaccharides to the surfaces of cell specific surface molecules and thus inhibiting the excess oxygen radicals (7) thus acting to alleviate the level of oxidative stress in the organism. Similarly, Su et al. (32) fed capsaicin also reduced MDA levels, which is consistent with the results of the present study, suggesting that the addition of capsaicin and MOP, a substance with a better biological function, can inhibit the serum levels of excess oxygen radicals in goat kids and protect the organism from oxidative damage.

Immune cells in animal bodies produce cytokines such as interleukins and anti-tumor factors, which have anti-disease functions. In this study, IL-2 and IL-6 had no significant effect among the groups, while TNF-*α* increased significantly with the increase of MOP addition. TNF-α is a protein produced by a variety of cells, and its main role is to regulate the immune response, promote inflammatory response, and regulate apoptosis (cell death) (33). When the organism is subjected to infection, tissue damage, or other pathological conditions, cells may release more TNF-α. Therefore, elevated levels of TNF-α in the blood or tissues are often considered as one of the indicators of inflammatory states. Elevated levels of TNF-α may indicate that the organism is experiencing an inflammatory response or immune system activation. However, high levels of TNF-α may also be associated with a number of other diseases and pathological conditions. Plant polysaccharides have been reported to improve phagocytosis by macrophages, leading to significantly higher levels of TNF-α (34, 35). It is possible that plant polysaccharides exert their antitumor effects by affecting tumor differentiation and apoptosis, altering intracellular signaling and immune regulation (36, 37). Immunoglobulins IgA, IgM, and IgG, as important immunoreactive molecules in animals, can specifically bind to the corresponding antigens and participate in the regulation of body immunity, so that the elevation of the levels of IgA, IgM, and IgG indicates the improvement of immune function. The addition of MOP significantly increased the immunoglobulin level in serum in this study, which is consistent with the results of Chen et al. (25), indicating that plant polysaccharides such as MOP have the effect of increasing immunoglobulin level to enhance the immunity of goat kids.

Chen et al. showed that pH in rumen fluid is closely related to VFA, and that an increase in rumen VFA concentration can lead to a decrease in rumen pH (25). This is similar to the results of the present study, where the VFA content was higher in the CON group than in the test group, while the pH was lower in the CON group than in the test group. In this study, according to the ratio of acetic acid to propionic acid, the majority of acetic acid fermentation could be judged. Meanwhile, the high level of green hay in the MOP group also proved that the cellulose in the rumen of goat kids was better decomposed to produce acetic acid. The energy produced by VFA in the rumen of lambs during its metabolism can directly stimulate the development of the rumen (38), with BA playing the most prominent role, followed by PA and AA. BA has been reported to have a promotional effect on the growth and development of rumen epithelial cells, and at the same time, it can reduce the apoptosis of rumen epithelial cells (39, 40). And the rumen epithelial tissue, as an important component of rumen function, has the function of transporting nutrients from the rumen to the bloodstream (40). IBA belongs to branched-chain fatty acids, which originate from the fermentation of protein feeds, and the fermentation process is accompanied by hazardous substances such as hydrogen sulfide and cresol, and a low amount of IBA can help to maintain the health of the rumen (24). VFA in the rumen is absorbed through the rumen epithelial cells, with an absorption rate depending on VFA concentration, rumen surface area and availability of transporter proteins (41). Another study showed that decreased serum GLU levels in young ruminants may be associated with incomplete development of the gastrointestinal tract (42). In this experiment, the serum GLU level, rumen papilla length density, muscularis propria thickness, and rumen wall thickness in the CON group were lower than those in the test group with MOP addition. Additionally, the IBA content in the test group was lower than in the CON group, suggesting that the rumen of MOP-added goat kids was more developed, the rumen environment was healthier, and VFA absorption was more efficient. The relative abundance of Succinivibrio and Megasphaera was higher in the CON group than in the MOP-added group. Since Succinivibrio is involved in the synthesis of short-chain fatty acids (43), this may explain why the PA, BA, and VA contents were higher in the CON group compared to the test group. This was further supported by the lack of significant difference in total protein content in serum among the groups. NH_3_-N in the rumen is mainly produced by fermentation of ingested protein feeds and is also an important source of microbial protein synthesis. Lv et al. demonstrated that the addition of fermented wheat bran polysaccharides selectively increased rumen microbial populations, which led to a decrease in NH_3_-N content and its uptake by the rumen epithelial cells (44). The results of the present study showed that the addition of MOP reduced the NH_3_-N content in the rumen of goat kids, and the lower NH_3_-N content may be a result of the efficient transport of NH_3_-N into the bloodstream by the better developed rumen epithelial tissues to participate in microbial protein synthesis, thus promoting the growth and development of goat kids.

The degree of rumen development can be measured by the development status of rumen epithelial cells, such as rumen papilla length and width (44). The rumen epithelium consists of rumen, muscularis propria, and epithelial cells, which have the functions of nutrient absorption, transportation, and metabolism (24). The fibrous component in the early primary goat kid diet is considered a key factor influencing the rumen development of goat kids. The fiber-rich diet provides the necessary physical stimulation for the physiological development of the rumen of young ruminants (45–47). At the same time, a large number of studies have shown that only after the rumen nipple start to grow and the thickness of the rumen wall begins to increase. Bian et al. (48) show that feeding fiber-rich alfalfa hay promotes the rumen peristalsis, accelerates the fermentation of rumen microorganisms, promotes the development of the rumen muscle layer, and further improves the digestive capacity of goat kids. In this study, the intake of goat kid mouth and green hay in the MOP test group was significantly higher than that in the CON group, and the addition of high-dose MOP significantly increased the height, muscle layer thickness and rumen wall thickness of the goat kids, indicating that the improvement of hay and open feed promoted the development of rumen in lactating ruminants. The proliferation of ruminal epithelial cells can also promote the growth of ruminal papilla and width and improve the muscle layer thickness. Other studies showed that increasing the height and width of the rumen nipple increased the absorption area of the rumen epithelium, but decreased the rumen nipple density (49). This is consistent with the results that adding MOP significantly reduced the density of rumen nipple in this study, but the addition of MOP had no effect on the width, cuticle thickness and specific surface area, which is inconsistent with Wang (24) and other studies, which may have different effects of different types of polysaccharide on the development of goat kids, and its regulation mechanism needs further study.

The rumen, as a specialized digestive organ in ruminants, contains a large number of microbial communities, such as bacteria, fungi, and archaea, which can help the host digest cellulose and other carbohydrates (50, 51). Microbial community colonization is also relevant and important for rumen development in young animals (52). Therefore, the effect of MOP addition on the structure and composition of rumen microbiota in goat kids was analyzed by 16SrRNA sequencing. In the present study, there was no significant difference between the CON group and the MOP test group in terms of flora alpha and beta diversity. Wang et al. (24). found that the addition of fermented bran polysaccharides did not have a significant effect on the alpha and beta diversity of the goat kid flora; Chen et al. (25) found that the addition of herbal polysaccharides did not have a significant effect on the alpha and beta diversity of the goat kid flora, which was in agreement with the present study. The current study showed that *Anaplasma* phylum, Thick-walled phylum and *Actinobacteria* phylum are the core phyla of the rumen with the highest relative abundance (1, 53). In this study, MOP had no effect on the structure and composition of the rumen microbiota. This might be due to the fact that the amount of milk replacer administered or its passage through the esophageal groove into the abomasum and intestines resulted in minimal impact on rumen fermentation and microbiota composition in goat kids (1). In addition, LEfSe analysis showed that goat kids in the LOW group were enriched with differential OTUs; most of these OTUs belonged to the genus Bacillus, and the Bacillus group includes many polysaccharide-degrading bacteria that contribute to the production of VFA in the gut. The cellulose secreted by Bacillus helps the animals to digest fiber, and produces various antimicrobial peptides that maintain the normal microbiota of the animals (54). Based on the present results and references, we hypothesize that MOP supplementation with MR stimulates the proliferation of fibrinolytic bacteria, such as Bacillus spp., which are producers of VFAs. This, in turn, increases the production of VFAs and microbial proteins, accelerates rumen development, and consequently improves the growth performance of early-weaned goat kids.

## 5 Conclusion

The addition of MOP to milk replacer powder in early-weaned goat kids increased daily weight gain, feed intake, immunoglobulin G, tumor necrosis factor *α* levels, and rumen height, while decreasing the levels of propionic acid, butyric acid, valeric acid, ammonia nitrogen, and density. Specifically, high doses of MOP (0.3%) significantly increased serum immunoglobulin A, immunoglobulin M, rumen muscle thickness, rumen wall thickness, and rumen contents pH, and also increased the relative abundance of Actinobacteria and Butyrivibrio species in the goat kid rumen. In summary, the addition of MOP to milk replacer powder for early-weaned goat kids can promote rumen growth and development, and improve immune function and growth performance.

## Data Availability

The data presented in the study are deposited in the Sequence Read Archive (SRA) repository, accession number PRJNA1159108.
