# Supplementary material for: Effects of moringa polysaccharides on growth performance, immune function, rumen morphology, and microbial community structure in early-weaned goat kids
Source: Front Vet Sci. 2024 Nov 6;11:1461391. doi: 10.3389/fvets.2024.1461391 (PMC11584012; doi:10.3389/fvets.2024.1461391)
Supplement: Supplementary file 1 [file Table_1.DOC]

Supplementary Material

# Supplementary Figures and Tables

Table 9 | Effect of MOP on the relative abundance of rumen microbiota at the phylum level (%)

| Items | CON | LOW | HIG | SEM | *p*-Value |
| --- | --- | --- | --- | --- | --- |
| Bacteroidetes | 39.44 | 38.10 | 44.84 | 3.04 | 0.676 |
| Firmicutes | 34.28 | 29.45 | 32.69 | 1.93 | 0.627 |
| Actinobacteria | 7.04 | 13.24 | 9.56 | 1.74 | 0.375 |
| Spirochaetes | 7.24 | 9.04 | 8.36 | 0.80 | 0.695 |
| Proteobacteria | 8.17 | 6.11 | 1.75 | 1.78 | 0.356 |
| Fibrobacteres | 3.02 | 2.04 | 1.03 | 0.80 | 0.638 |
| Tenericutes | 0.16 | 0.40 | 0.34 | 0.11 | 0.697 |
| Verrucomicrobia | 0.06 | 0.14 | 0.28 | 0.09 | 0.645 |
| Cyanobacteria | 0.02 | 0.02 | 0.04 | 0.00 | 0.457 |
| Synergistetes | 0.02 | 0.03 | 0.02 | 0.00 | 0.344 |
| Others | 0.54 | 1.43 | 1.02 | 0.33 | 0.595 |

CON, the milk replacer (n = 4); LOW, supplemented with 0.15% MOP in the milk replacer (n = 4); HIG, supplemented with 0.3% MOP in the milk replacer (n = 4).

Table 10 | Effect of MOP on the relative abundance of rumen microbiota at the genus level (%)

| Items | CON | LOW | HIG | SEM | *p*-Value |
| --- | --- | --- | --- | --- | --- |
| *Prevotella* | 24.88 | 19.71 | 27.82 | 1.91 | 0.228 |
| *Olsenella* | 4.09 | 11.05 | 7.58 | 1.44 | 0.140 |
| *Ruminococcaceae_Ruminococcus* | 5.39 | 9.06 | 5.24 | 1.20 | 0.372 |
| *Sharpea* | 6.39 | 3.73 | 7.71 | 1.52 | 0.600 |
| *Treponema* | 5.03 | 6.71 | 4.98 | 1.05 | 0.780 |
| *Succinivibrio* | 7.50 | 2.08 | 1.11 | 1.52 | 0.188 |
| *Sphaerochaeta* | 2.21 | 2.32 | 3.38 | 0.73 | 0.802 |
| *Butyrivibrio* | 1.93 | 2.70 | 2.60 | 0.67 | 0.900 |
| *Fibrobacter* | 3.02 | 2.04 | 1.03 | 0.80 | 0.638 |
| *Megasphaera* | 4.02 | 1.31 | 0.32 | 1.34 | 0.552 |
| *Succiniclasticum* | 2.18 | 1.13 | 0.99 | 0.28 | 0.159 |
| *Ruminobacter* | 0.05 | 3.61 | 0.00 | 1.20 | 0.408 |
| *YRC22* | 0.38 | 1.96 | 0.29 | 0.51 | 0.356 |
| *Anaerovibrio* | 0.91 | 0.32 | 0.74 | 0.15 | 0.303 |
| *Schwartzia* | 1.22 | 0.13 | 0.19 | 0.34 | 0.365 |
| *Others* | 30.81 | 32.14 | 36.01 | 2.41 | 0.701 |

CON, the milk replacer (n = 4); LOW, supplemented with 0.15% MOP in the milk replacer (n = 4); HIG, supplemented with 0.3% MOP in the milk replacer (n = 4).
